# Non-Targeted LC-MS Metabolomics Approach towards an Authentication of the Geographical Origin of Grain Maize (*Zea mays* L.) Samples †

**DOI:** 10.3390/foods10092160

**Published:** 2021-09-13

**Authors:** David Schütz, Elisabeth Achten, Marina Creydt, Janet Riedl, Markus Fischer

**Affiliations:** 1 Hamburg School of Food Science, Institute of Food Chemistry, University of Hamburg, Grindelallee 117, 20146 Hamburg, Germany; david.schuetz@uni-hamburg.de (D.S.); marina.creydt@uni-hamburg.de (M.C.); 2 Department Safety in the Food Chain, German Federal Institute for Risk Assessment (BfR), Max-Dohrn-Str. 8-10, 10589 Berlin, Germany; elisabeth.achten@gmail.com (E.A.); janet.riedl@bfr.bund.de (J.R.)

**Keywords:** grain maize, LC-MS(/MS), geographical origin, authentication, metabolomics

## Abstract

Safety along the food and feed supply chain is an emerging topic and closely linked to the ability to analytical trace the geographical origin of food or feed. In this study, ultra-performance liquid chromatography coupled with electrospray ionization quadrupole-time-of-flight mass spectrometry was used to trace back the geographical origin of 151 grain maize (*Zea mays* L.) samples from seven countries using a high resolution non-targeted metabolomics approach. Multivariate data analysis and univariate statistics were used to identify promising marker features related to geographical origin. Classification using only 20 selected markers with the Random Forest algorithm led to 90.5% correctly classified samples with 100 times repeated 10-fold cross-validation. The selected markers were assigned to the class of triglycerides, diglycerides and phospholipids. The marker set was further evaluated for its ability to separate between one sample class and the rest of the dataset, yielding accuracies above 89%. This demonstrates the high potential of the non-polar metabolome to authenticate the geographic origin of grain maize samples. Furthermore, this suggests that focusing on only a few lipids with high potential for grain maize authentication could be a promising approach for later transfer of the method to routine analysis.

## 1. Introduction

The traceability of goods, especially food and feed, is an emerging topic, as supply chains are more and more complex in a globalized market. Today the verification is mainly done by checking shipping documents, which may be falsified. Methods such as the blockchain strategy, which in principle make it possible to trace the entire supply chain back to its origin, are being developed, but are not immune to manipulation from the real world (from the field) into the digital world (the blockchain). In short, if the grower provides false information, this information is passed on without further verification. Blockchain technology simply ensures that these data, whether correct or incorrect, can no longer be manipulated within the chain. The only way to verify this is by using experimental analytical methods [1]. Therefore, there is a high demand for analytical methods to objectively verify the origin of feed or food samples [2].

Traditional analytical methods of cereal-based food or feed focus on specific quality parameters such as moisture, protein, oil and starch content [3,4] but also contaminants such as mycotoxins [2,5]. Verification methods for origin determination often use spectroscopic or spectrometric instruments for analysis [6,7]. Unlike conventional methods, such a non-targeted approach uses the entire spectrum of data that can be collected to determine authenticity [6]. Analysis of the metabolome for origin verification is particularly suitable because it is closest to the phenotype and therefore sensitive to environmental influences [8]. The usage of gas or liquid chromatography coupled with mass spectrometry (LC-MS) is common [7,8,9], as well as the usage of nuclear magnetic resonance spectroscopy [6,9]. Differences in the fatty acid profile of various matrices were often observed in investigations related to the verification of the origin of feed and food [10,11]. Especially in the case of corn-based distillers dried grains with solubles (DDGS), it was found to be suitable for authentication purposes [12]. These results suggest that the lipid fraction in particular may contain promising markers for geographic origin verification. Indeed, the non-polar metabolite fraction, has also been shown to be suitable in high resolution metabolomics-based food origin verification studies using LC-MS techniques [13,14,15,16,17].

Due to the huge amount of data, data analysis in non-targeted metabolomics studies is often performed using multivariate data analysis, which reduces the high dimensional data and facilitates the observation of differences [7,8,9,18]. Classification algorithms can be applied based on the observed pattern to predict new samples [6,7,19]. The measured patterns of samples, also called “fingerprints”, can be used to develop a targeted method that does not require high-resolution instrumentation. At the same time, it allows routine comparisons of molecular patterns of unknown samples with reference datasets stored in the database [6,14].

Maize (*Zea mays*, L.) is an important cereal that is widely used as feed material, especially for poultry, but also as food for humans and in the renewable energy and bioethanol production [3,20]. The market for maize is internationalized, with over 15% (175 million tons) of the world’s maize production volume of more than 1116 million tons exported or imported in 2019/20, making it an ideal demonstration matrix for feed origin verification [21]. Overall, geographic origin can affect the quality of grain maize, which can also lead to different risks of mycotoxin contamination [2]. Consequently, it is important to develop methods to verify the geographical origin, not only because of possible food or feed fraud, but also, and above all, because of safety along the supply chain.

Corn and other cereals such as wheat, oat and barley are often examined by LC-MS to determine mycotoxins [5]. In contrast, they were rarely studied regarding their authenticity using mass spectrometry-based methods, which were mostly gas chromatographic approaches [12,22,23]. There were also some approaches to study breeding results of maize utilizing LC-MS, which showed that differences in the metabolome of grain maize and forage were more altered by geographical origin than by varietal influences [24,25].

Therefore, the aim of this study was to analyze the metabolome of grain maize used as feed material or for starch production, in particular the lipidome, by means of ultraperformance liquid chromatography coupled with high resolution electrospray ionization quadrupole-time-of-flight (UHPLC-ESI-qToF) mass spectrometry, to reveal differences between the origin-specific sample groups of a sample set covering seven countries worldwide. Variation of the analytical data, particularly with respect to growth origin, was evaluated using multivariate data analysis. Highly potent marker metabolites for growth origin verification that were both significant and reliable were further identified by LC-MS/MS fragmentation. These markers were then used in classification models based on the Random Forest (RF) algorithm to verify their usability for geographic authentication. To account for the fact that genomic variability could bias this analytical approach, a portion of the samples were from different cultivars but grown at the same location.

## 2. Materials and Methods

### 2.1. Reagents and Chemicals

Acetonitrile (LC-MS grade), ammonium formate (≥95%), chloroform (HPLC grade), isopropanol, methanol (both LC-MS grade) and sodium hydroxide (≥99%) were purchased from Carl Roth (Karlsruhe, Germany). (1*H*,1*H*,2*H*-perfluoroethoxy)phosphazene was obtained from Santa Cruz Biotechnology (Dallas, TX, USA). Ultrapure water (resistance ≥ 18 MΩ) was obtained by treating demineralized water in a Direct-Q 3 UV-R system (Merck Millipore, Darmstadt, Germany).

### 2.2. Grain Maize Samples

The sample set consisted of a total of 151 samples: 19 from Ukraine, 25 from France (region Alsace), 25 from Hungary, 14 from Slovakia, 20 from Spain, 25 from Peru (region Lambayeque) and 23 from the United States of America (USA). Samples from France and Hungary were intended for starch production, while the other samples were intended as feed material. In order to take into account the influences of varieties on the metabolome, the samples from Spain came from a varietal trial, with every single sample belonging to a different variety; see Tables S1 and S2 for more details about the samples’ metadata. Samples from the USA were supplied with extended geographical information. Four samples each came from Alabama, Arkansas, Georgia, Louisiana, Mississippi and three from Texas. For quality control, a commercial batch of grain maize from Germany was used. A part of this sample set was also analyzed by Achten et al. by means of Fourier transform infrared spectroscopy [26].

### 2.3. Sample Treatment

Grain maize samples were ground in an ultra-centrifugal mill (ZM 200, mesh size 0.5 mm, Retsch GmbH, Haan, Germany) and homogenized in a drum hoop mixer (3 h at 28 rpm, RRM 100, J. Engelsmann AG, Ludwigshafen, Germany). A part of the samples (origin France and Hungary) was ground in a Grindomix GM 300 knife mill (Retsch, Haan, Germany) with addition of the same amount of dry ice, after being flash frozen in liquid nitrogen.

The ground samples were then lyophilized for 24 h (Beta 1-8 LDplus, Christ, Osterode, Germany) and stored at −21 °C until further analysis. 50.00 ± 0.25 mg of the lyophilisate was extracted using a BeadRuptor24 ball mill (Omni International, Kennesaw, GA, USA), by a variation of the Bligh and Dyer lipid extraction method, as used previously by Creydt et al. for the extraction of non-polar metabolites of plant sample material [13]. First two steels balls, and 750 µL of a mixture of chloroform/methanol (2/1, *v*/*v*) were added. Subsequently, sample disruption was done for 1 min using the ball mill. Then 250 µL chloroform and 500 µL water were added and extracted with the ball mill for 2 min. The suspension was centrifuged at 16,000× *g* for 3 min at 4 °C to induce phase separation. The lower, organic phase was diluted by factor 1:5 with isopropanol/acetonitrile (3/1 *v*/*v*) and centrifuged for 10 min, at conditions mentioned above. The supernatant was then subjected to LC-MS resp. LC-MS/MS analysis.

### 2.4. LC-MS(/MS)-Analysis

Liquid chromatography was performed on a Dionex UltiMate 3000 UPLC System (Thermo Fisher Scientific, Braunschweig, Germany). An Accucore RP-MS UPLC column (150 mm × 2.1 mm i. d., 2.6 μm particle size), equipped with a guard column of the same material (10 mm × 2.1 mm i.d., Thermo Fisher Scientific, Braunschweig, Germany) was used to separate the metabolites at 30 °C with a flow rate of 300 μL/min. The injection volume was 8 µL. Water was used as mobile phase A. Mobile phase B consisted of isopropanol/acetonitrile (3/1, *v*/*v*), both containing 10 mmol/L ammonium formate as additive. Gradient starting conditions were 55% B, at minute 2 increasing linearly to 75% at minute 4, then to 100% at minute 18. Then holding 100% B for 4 min, and then going back to starting conditions (55% B) in 1 min. The total chromatography method length was 27 min.

Mass spectra were acquired on an ESI-qToF mass spectrometer (maXis 4G, Bruker Daltonics, Bremen, Germany). Measurements were done in positive ion mode, ranging from *m*/*z* 80 to 1000. The ESI source conditions were as followed: Capillary voltage 4500 V, end cap offset: 500 V, nebulizer gas 4 bar, dry gas 9.0 L/min, drying temperature 200 °C. Calibration of the mass spectrometer was done by direct infusion of sodium formate cluster solution before measurements and also at a flow rate of 0.1 mL/h at the minute 26 of each run by means of a switching valve and a syringe pump. Additionally, a solution of 1 mg/mL (1*H*,1*H*,2*H*-perfluoroethoxy)phosphazene dissolved in isopropanol was applied as lock mass. Fragmentation spectra were obtained by collision-induced dissociation mode using collision energies of 20, 40 and 60 eV. Mass spectrometry detection and LC gradient conditions were used as optimized by Creydt et al. and applied to asparagus origin determination sample set [13]. To avoid false discovery of marker substances due to instrumental drift, all samples were measured in one batch in random order. To assure the reliability of measurements, a quality control (QC) sample (geographical origin Germany), was extracted alike the analyzed origin samples and was repeatedly injected after every 12 origin samples.

The selected marker substances were further identified by their characteristic fragmentation patterns obtained by LC-MS/MS analysis and manual structure elucidation, lipid class verification and identification of linked fatty acid chains. In addition, a comparison of the resulting fragmentation patterns with database deposited fragmentation spectra/schemes in the Human Metabolome Database (HMDB) or LipidMaps databases (“MSAnalysis Tool”) was conducted to verify substance identification [27,28,29,30].

### 2.5. Data Processing

Mass calibration and feature detection for each analysis file were done in DataAnalysis 4.1 software (Bruker Daltonics, Bremen, Germany). Parameters passed to the function “Find Molecular Features” were: signal-to-noise threshold of 5, correlation coefficient of 0.7, minimum compound length of 8 and a smoothing width of 2. Adducts considered were [M + H]^+^, [M + Na]^+^, [M + K]^+^, [M + NH_4_]^+^, [M − H_2_O + H]^+^, [M − CO_2_ + H]^+^, [M − NH_3_ + H]^+^.

Collection of molecular features was done using Profile Analysis 2.1 software (Bruker Daltonics, Bremen, Germany). A consensus table was generated by using the advanced bucketing function, which grouped features by *m*/*z*-value and retention time in “buckets”. Parameters for collecting consensus features were: ΔRT = 20 s and Δ*m*/*z* = 0.02 Da. A time alignment was conducted and features, which appear in the bucket table, were set to be detectable in 50% of all sample analyses. Missing values of features were replaced by the lowest detected level in all samples. Consensus peak heights were then exported as csv-files and reformatted in Microsoft Excel 2010 for subsequent multivariate data analysis in R version 3.6.3 [31].

The first assumption of identification was made by looking up *m*/*z*-values in metabolite databases (HMDB [27] and LipidMaps [28]). Peak areas of promising metabolites were determined by integration of extracted ion chromatograms (EIC) of the [M + H]^+^-peak in case of phospholipids or the [M + NH_4_]^+^-peak for diglycerides (DG) resp. triglycerides (TG). At this stage of the analysis, mainly the [M + H]^+^ resp. [M + NH_4_]^+^ adduct peaks were used for integration, since sodium or potassium ions were only present in small, rather non-reproducible amounts due to contamination [32].

EICs were obtained from the calibrated data files by using QuantAnalysis 2.1 software (Bruker Daltonics, Bremen, Germany). The extraction window was set to ± 0.3 Da. Then peak areas were obtained by integration of peaks at the corresponding retention time ± 0.5 min. Peak area data were also exported as csv-files and reformatted in Microsoft Excel 2010 for further visualization using boxplots and statistical analysis, both conducted by using R. Used function calls are given in the supplement.

General statistical significance of multi class comparisons was accessed by one-way analysis of variance (ANOVA) test with a significance level of 0.01. The significance on country level was further examined by a post-hoc Tukey-test. The least significant differences (LSD) between the sample classes at raw data level were determined using the *LSD.test* function from the R package agricolae [33], with a significance level of 0.01 using the Bonferroni correction. The relative standard deviation (RSD) of metabolites was calculated from peak areas of ten QC samples.

### 2.6. Multivariate Data Analysis and Classification

Principal component analysis (PCA) was conducted in R, after pareto scaling, by using the *prcomp* function. The importance of features for separation in the PCA scores plot was estimated by inspecting their position in the PCA loadings plot of the first three principal components. Mahalanobis distances were calculated for each group on the basis of pareto scaled data by the *mahalanobis.distance* function from the R package HDMD [34]. Canonical variate analysis (CVA) was conducted after pareto scaling using the *lda* function from the R package MASS [35]. Multivariate ANOVA (MANOVA) was conducted on the first three principal components using the *manova* function in R, employing the Wilk’s test.

Classification was performed on pareto scaled data by using the RF algorithm with 1000 trees, contained in the R package randomForest [36]. The classification models were validated in 100 times repeated 10-fold cross-validation (CV) by the *train* function of the caret package [37]. Sample classes were the geographical origin of the samples. In case of one vs. rest models one classification model was built per sample class (in total 7 models). In each model, one geographical origin was used as one class, while the remaining samples in the dataset were assigned to the other class “rest”, regardless of their growth origin (binary classification). Classification results were reported as accuracy, along with its standard deviation over all repeats of CV. Detailed confusion values were reported as mean values over the 100 repeats of 10-fold CV. Variable importance during classification was obtained by the function *varImp* included in the caret package.

## 3. Results and Discussion

### 3.1. General Evaluation of the Analytical Method

In this study, 151 individual grain maize samples from seven countries (14–25 samples/country) worldwide were measured by LC-MS to establish a model for analytical geographic origin determination of commonly traded grain maize. A representative analysis of a grain maize sample by LC-MS is shown in Figure S1. Already during inspection of the mass spectrometry raw data, it became obvious by matching *m*/*z*-values (Δ*m*/*z* ≤ 5 ppm) to those in the HMDB database [27], that the present peaks were mostly lipids. These findings are in accordance with the used non-polar Bligh and Dyer extraction method. Regarding lipid classes, phospholipids (especially phosphatidylcholines (PC) and phosphatidylethanolamines (PE)) as well as acylglycerides (DGs and TGs were present. DGs and phospholipids were both eluting in the retention time range of 10.5–18.5 min. However, DGs ranged from *m*/*z* 580–750, while phospholipids tend to be observed at higher *m*/*z*-values (*m*/*z* 700–850). TGs eluted almost entirely separately at retention times >17.5 min. Found numbers of peaks related to certain substance groups were in decreasing order: TG >> DG > PC > PE. Beside these species, a few monoglycerides, free fatty acids and predominantly low molecular features occurred, mostly in-source fragments [acyl + 74]^+^ of DGs or TGs [38]. DGs and TGs occurred mainly as ammonium adducts ([M + NH_4_]^+^), but also as sodium adducts ([M + Na]^+^), following literature [29]. In lower abundance phospholipids appeared; PCs and PEs mainly as [M + H]^+^ ions and PCs also as prominent sodium adducts [30].

### 3.2. Multivariate Data Analysis

**Figure 1:**
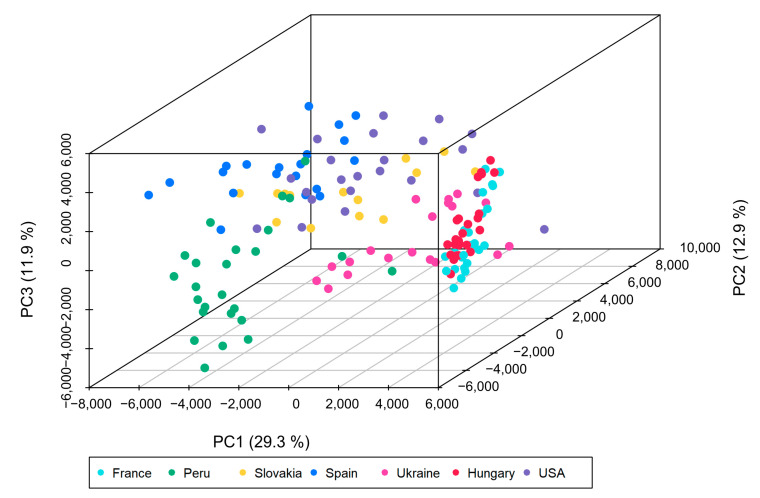
Principal component analysis (PCA) scores plot created using all features of the consensus peak table, after pareto scaling as preprocessing. The first three principal components (PC1-PC3) are displayed.

To get an overview of the ability to distinguish by means of LC-MS analysis data between the different geographical origins in the grain maize sample set, the peaks of all analysis in the dataset were matched. This resulted in a consensus peak table containing 1022 features (see Appendix A), which was subjected to PCA. A PCA reduces the dimensionality of the data, while preserving the variance in the dataset. The resulting scores plot of the first three principal components is displayed in Figure 1.

In the PCA scores plot, clustering of the samples according to the origin of cultivation was achieved, indicating that the values of the features determined by LC-MS analysis of the grain maize samples vary along the geographical origin. The explained variance using only three principal components was not very high (six principal components were needed to explain ≥95% cumulative variance); although the samples from each origin were clustered together. From the PCA scores plot, it was observed that the samples from the USA, Slovakia and Spain were relatively close. The proximity of the USA samples to parts of the European samples is initially surprising, but since these samples came from different locations of the USA with varying climatic conditions, the behavior is conceivable. A second cluster consists of samples from France, Hungary and Ukraine, which is consistent with Central Europe origins. Samples from Peru cluster separately from the other sample groups, as would be expected based on geographic origin.

In addition, a CVA was conducted to analyze the sample set especially for differences between the different sample classes (geographical origin). The CVA scores plot is shown in Figure S2. Based on the discriminant function, the samples formed clusters according to their geographical origin, as expected. Samples from France/Hungary were relatively close to each other and separated from the other samples. Samples from the USA and Spain were also relatively close to each other, but still formed clusters according to geographical origin. Samples from Peru, Hungary and Slovakia formed distinct clusters, well separated from the samples from other geographical origins. MANOVA conducted for a combination of the first three principal components showed that the result for the second and third principal components was significant (*p*-value ≤ 0.01, for further statistical results see Table S3). Inspection of the PCA loadings plot revealed features that were responsible for clustering samples by geographic origin in the scores plot. Promising features belong mainly to the compound classes of TGs (e.g., *m*/*z* 932.9 ([M + NH_4_]^+^ adduct of TG (56:2)) or *m*/*z* 868.7 ([M + NH_4_]^+^ adduct of TG (52:6)) and phospholipids (e.g., *m*/*z* 784.6 ([M + H]^+^ adduct of PC (36:3) or *m*/*z* 716.5 ([M + H]^+^ adduct of PE (34:2)), but also DGs (e.g., *m*/*z* 637.5 ([M + Na]^+^ adduct of DG (36:5)).

### 3.3. Identification of Relevant Marker Substances

Non-targeted metabolomics studies provide a very large number of features. Although not all of them may be suitable for the intended purpose of geographic origin differentiation, there is a need for reducing the number of features used, if they are also to be suitable for use in routine setting. This is even more important because the ToF instruments used are very expensive and comparatively sensitive. Downstream analysis is often performed by other instruments such as triple quadrupole mass spectrometers in multi reaction monitoring mode [1]. In addition, short chromatographic runs are goal-orientated, so that the maximum of substances detected in parallel should not be too high in order to avoid ion suppression effects [39]. The reduction in the number of features should be done in such a way that accuracy of the origin discrimination is maintained, although the number of features used is significantly reduced. Therefore, powerful markers for origin determination of grain maize were selected to be both reliable and informative at the same time. The markers used had to meet the following acceptance criteria:

1. Show significance in one-way ANOVA-test regarding the geographical origin (*p*-value of ≤0.01);
2. Have a high count of >7 out of 21 possible significant differences between country pairs determined by post-hoc Tukey test;
3. Exhibit a RSD below 15%.

To obtain a balanced model that has a good performance for correctly predicting each included sample class, additional care was taken to ensure that each selected marker substance was significantly different in the Tukey-Test for separation between each included sample class and at least one other country.

In order to increase the robustness, a combination of marker substances from all four lipid classes (PC, PE, DG and TG) that were prominent in the grain maize samples (see Section 3.1) was selected. To further facilitate the analytical measurement, substances with higher abundance were preferred.

**Figure 2:**
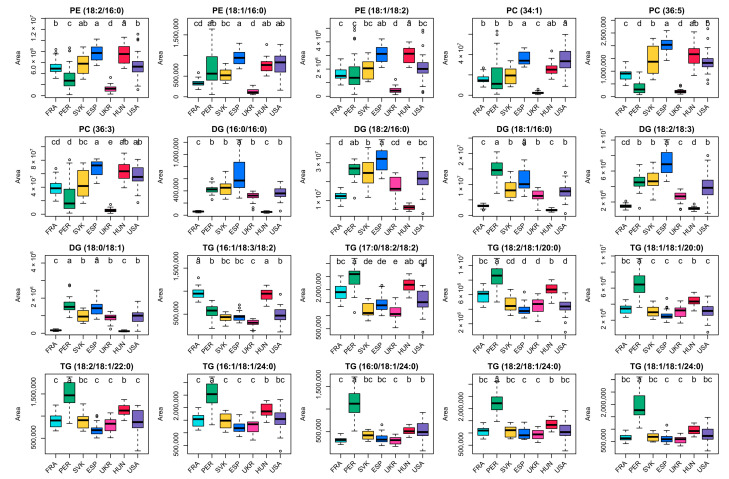
Boxplots of peak areas per sample origin of promising marker substances for the origin verification of grain maize samples. Differences between samples classes are significant (*p*-value ≤ 0.01), if labeled with different letters; for all *p*-values see Figure S3.

This allowed the identification of 20 possible marker substances, all of which showed a *p*-value ≤ 0.01 in a one-way ANOVA-test with respect to the geographical origin (significance level 0.01) and a RSD in QC samples ranging from 2.6–9.3% (median: 5.4%). The maximum number of country pairs that each marker substance could be distinguished (revealed by post-hoc Tukey test) was 16 (achieved by four markers) and the median was estimated to 12.5. From the country perspective, the French, Peruvian, and Hungarian samples had relatively many metabolites with significantly different metabolite values to distinguish them from the other countries; in contrast, the Slovakian samples were the most difficult to distinguish from the other countries. These results were also reflected in the RF classification results (see Section 3.5). The exact identities of the marker substances (as evident by LC-MS/MS fragmentation analysis, see Section 3.4) are given in the boxplot (see Figure 2); additional statistical results are shown in Table S4.

It was notable that peak areas differed between growth origins, but no systematic differences of all considered markers were observed in one sample origin. The detected values of some classes of marker in certain countries were striking: the low levels of all phospholipid markers in Ukrainian samples and the low levels of most DG marker substances in French and Hungarian samples. These samples were not used as feed but as raw material for starch production and were homogenized in the frozen state with a slightly different protocol using a knife mill and dry ice. With the exception of DGs, the peak areas for all other metabolites analyzed were in a comparable range to the other samples used as feed material. Since milling with the addition of dry ice is also known as to be gentler on the metabolite-state [1], the low DG content is presumed to be related to the use case of starch production. Nevertheless, the selected metabolites were potent markers also for a geographical distinction between the origins of France and Hungary. 9 of the 20 metabolites differed significantly by LSD results (*p*-value ≤ 0.01) between French and Hungarian samples: all PE markers, PC (36:5), PC (36:3), DG (34:2), TG (58:3), TG (58:2) and TG (58:1). In relation, high levels of marker substances were observed for: Phospholipids in samples from Spain and Hungary, DGs in Spanish samples and TGs in Peruvian samples. The high concentrations of TGs in samples from Peru could be related to the particular growing region, located on the one hand nearby the equator and on the other hand in a coastal region with proximity to the Andes.

Reasons for the different regulation of lipid classes could be that TGs are storage lipids, while DGs are playing an intermediate role in the lipid biosynthesis pathway [40]. In contrast, phospholipids are membrane lipids whose regulation is often associated with environmental factors such as temperature, soil composition, drought, pathogens and others [40,41], which in turn may be related to the geographic origin. Therefore, higher variation of phospholipid levels compared to the other two lipid classes is conceivable due to environmental differences. In general, plants respond to stressors such as changing climatic conditions by altering membrane fluidity by modulating the extent of fatty acid unsaturation [41,42]; in a more special case, it was shown that maize leaves react to temperature stress by regulating lipids [43].

There are some approaches in literature to show the relationship between genetics and oil content of maize plants [44]. Laurie et al. found that multiple quantitative trait loci (QTL) are involved in the regulation of oil synthesis in maize [45], which could complicate the breeding of varieties with high oil content. Yang et al. correlated QTL with fatty acid content and de Abreu e Lima et al. linked the results of lipidomics experiments to QTL, to show the relationship between lipid content and genes involved in lipid biosynthesis [44,46]. The high number of genes involved in the regulation of oil synthesis protects to some extent the lipid-based approach to authenticate the geographical origin with respect to varietal influences. Among the selected TG markers, a preference for linoleic acid FA (18:2) and oleic acid FA (18:1) can be seen in the marker molecules, which is consistent with the high occurrence of these fatty acids in maize oil [44].

General variabilities in the fatty acid profile measured by gas chromatography in maize and wheat DDGS were also used to differentiate geographic origin by Tres et al. [12]. Regarding the verification of the geographical origin of other plant matrices using lipid species measured by LC-MS-based analysis, Lim et al. as well as Rubert et al. also found that phospholipids in particular were able to differentiate between different geographical origins of samples in non-targeted metabolomics studies on the geographical origin of rice and tiger nuts, respectively [15,17]. Phospholipids, DGs and TGs were also found to be able to differentiate the geographical origin of hazelnuts [14]. In addition, glycerolipids and glycerophospholipids in particular were found to be potential marker substances in a study on the possibility of verifying the geographical origin of Chinese pork samples [16].

Phospholipid and acylglyceride biosynthesis are closely linked. Differences in lipid biosynthesis may be caused by exogenous factors as well as by a different genetic background of plants [40]. This may influence the lipid profile, which could affect the concept of verifying the geographical origin based on lipid species. To address these concerns, the samples from Spain were from a varietal trial consisting of 20 different maize varieties. Interestingly, most of the selected DGs showed a higher variation within the Spanish sample class than in the other classes, suggesting that the DG content might be influenced by the grain maize variety. Nevertheless, the Spanish samples of different varieties did not show an obviously different clustering in the scores plot of the PCA than the other samples, nor did they show a worse result in the RF classification (>19 of 20 Spanish samples correctly classified, see Section 3.5). These results also followed previous findings of Baniasadi et al. and Tang et al., which also showed that the influence of genetic vs. environmental factors on the metabolome of grain maize samples is much more on the environmental side than varietal factors [24,25]. Therefore, the results can be seen as indicator that the genomic background of grain maize cultivars commonly used as feedstuff has no negative influence on the classification model for origin verification based on lipid markers.

### 3.4. Structural Elucidation of Marker Metabolites

A first evidence of substance identity was already that the peaks used had matched *m/z*-values with values deposited in metabolite databases (HMDB, LipidMaps [27,28]). Further structural elucidation was then performed by recording LC-MS/MS spectra and obtaining structural information from fragmentation patterns. Relevant structural information was derived from specific fragment pattern present in the MS^2^-spectra. The observed fragment ions were manually compared with the fragmentation schemes deposited in the databases. In particular, for PCs, an abundant peak was observed in the MS^2^-spectrum at *m*/*z* 184, corresponding to the formation of a phosphocholine ion [C_5_H_15_NO_4_P]^+^ from the polar head group [30]. In addition, the corresponding [M − 183 + H]^+^ fragment was detected with very low intensities. In the same way, PEs were identified by a fragment peak [M + H − 141]^+^, corresponding to the loss of a phosphatidylethanolamine head group (C_2_H_8_NO_4_P). Further neutral loss of one fatty acid chain leads to a fragment ion [RCOOCH_2_COCH_3_ + H]^+^ [30]. The presence of DGs or TGs was indicated by the appearance of prominent fragment ions caused by the loss of one fatty acid chain and one ammonia molecule [M + NH_4_ − (RCOOH + NH_3_)]^+^ as well as the appearance of [RCO]^+^ ions [29]. Identification of the fatty acid chain lengths and count of double bonds for PE and DG lipids was conducted by observation of the occurrence of [RCO + 74]^+^ ions. For TGs, the fatty acid chain pattern was elucidated by the ions generated by neutral loss of one of the fatty acid chains and ammonia. The structure of PCs could not be further resolved at the fatty acid chain level, due to the intense phosphocholine peak at *m*/*z* 184. The specific fragments in the MS^2^-spectra, which led to their identification as lipids, are noted along with structural information in Table S5.

Fourteen of the marker substances belong to the class of acylglycerides, nine were TGs and five were DGs. Six substances belong to the substance class of phospholipids (three each were PCs resp. PEs). Most of the identified lipids consist only of even-numbered fatty acid chains. Since acyl coenzyme A is subsequently formed by repeatedly adding acetyl coenzyme A to the growing acyl chain, the fatty acid residues tend to have an even number of carbon atoms [40]. However, there were also some lipids present in the grain maize samples containing odd-numbered fatty acid chains (e.g., TG (17:0/18:2/18:2)), but in much lower concentrations than even-numbered fatty acid residues. The occurrence of various odd-numbered lipids in plants has been shown in the past [47,48].

### 3.5. Further Investigation of the Identified Potent Marker Substances

**Figure 3:**
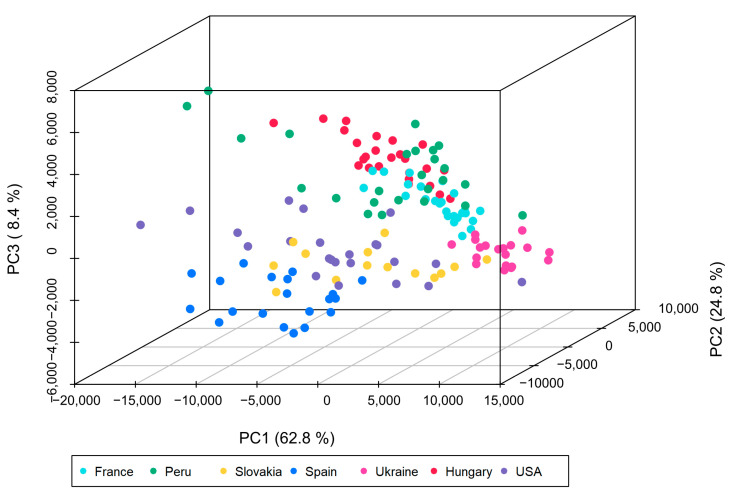
PCA scores plot created using peak areas of only the 20 high potential marker substances, after pareto scaling as preprocessing.

The PCA scores plot (Figure 3) shows the partitioning of the samples based on only the set of 20 identified compounds. The raw peak area data of the markers in the grain maize sample set is given in Appendix B. It could be observed that the samples were again clustered according to their country of origin, in the PCA scores plot. The variance explained by the first three principal components was 96%. At the country level, the dense clustering of samples from Hungary and France was improved by reducing the number of features used for classification. In addition, the clustering of Ukrainian and Spanish samples improved significantly. The samples from Peru changed their location and are now closer to the samples from France and Hungary. Differentiation of samples from the USA and Slovakia remains a challenge. Mahalanobis distances were calculated to quantify the differences; the results matrix is displayed in Table S6.

MANOVA analysis showed that the differences between the geographical origins were significant (*p*-value ≤ 0.01, for further statistical results, see Table S3). Again, a CVA was conducted; the scores plot is shown in Figure S4. Compared to the results of the CVA with all metabolites detected, the separation of the samples according to the geographical origin was similar, when only 20 lipids were used for differentiation. All samples were well separated in clusters, although the samples were less close together, then when all features were used.

To evaluate the ability of the selected high potential marker substances to predict the geographical origin of grain maize samples, a classification model using the RF algorithm was calculated. To assure that the model was not biased due to overfitting to the sample set, the classification was conducted as 10-fold CV, which was repeated 100 times to ensure reliability as well as to estimate the standard deviation. RF classification led to an overall classification accuracy of 90.5 ± 1.4%. In relation to the classification accuracy obtained by using all features of the consensus peak table (98.1 ± 0.3%), a comparably accurate result was obtained, although the number of features used was greatly reduced by a factor of about 50 (only 20 out of 1022).

**Figure 4:**
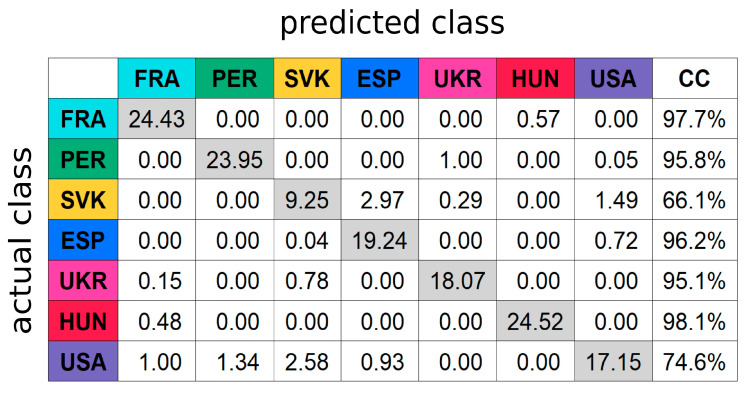
Mean Random Forest (RF) classification matrix using peak areas of 20 marker substances (after pareto scaling). The confusion values were given as average of 100 repeats of 10-fold cross-validation (CV) along with correct classification (CC) rates.

Looking at the classification performance at the country-level (see Figure 4), the classification results are consistent with the clustering of the samples in the PCA scores plot. Samples from Peru, Spain, France, Ukraine and Hungary are excellent at classification (correct classifications (CC) > 95%). Meanwhile, samples from Slovakia and the USA are still challenging for the model to separate (CC 66% and 75%, respectively); some Slovakian samples were also misclassified as Spanish. Samples intended for starch production (geographical growth origin France resp. Hungary) were completely separated from the other samples used as feed material in the classification matrix. Nevertheless, a high success rate in the classification of samples from both origins France and Hungary (CC > 97%) indicates that the selected marker substances can also be used in a context of geographical authentication of grain maize samples for starch production.

Specifically for the samples from the USA, a greater diversity could also be observed in the sample point cloud in multivariate data analysis (as well as by RF results). This greater variance could be explained by the fact that the samples from the USA were distributed over six states in the southern of the U.S., covering an east-west distance (Texas-Georgia) of approx. 1400 km and a north-south distance of approx. 900 km. Compared to samples of other geographical origins such as Spanish, Slovakian but also Peruvian samples, the U.S. samples were geographically more widely distributed. Comparing the classification accuracies with those obtained in the literature, comparable results were achieved: Tres et al. who authenticated maize DDGS samples from two regions in China and the USA by means of the fatty acid profile, achieved comparable accuracies per sample class by partial least squares discriminant analysis classification models [12].

In addition, it must be clarified that the occurrence of different concentrations of marker substances does not consider national borders, but is based on many different influencing factors, e.g., soil composition, climate conditions: solar radiation, water ability, but also usage of fertilizers and pesticides etc. [9]. These influencing factors may be more likely to differ between samples grown at a greater geographic distance than between samples grown in close proximity to each other.

The ability of the selected marker set was further analyzed by calculating binary one vs. rest RF classification models for each geographical origin included in the sample set. These models had to classify the samples to be either from one specific country or from the other countries (summarizing all other sample origins included in the dataset as one class). Thereby, classification accuracies of above 89% could be achieved (see Table 1).

The separation of the Hungarian samples from all other samples in the dataset did perform well (above 99% accuracy), while the separation of samples from the USA did perform worse with only 89% accuracy. The classification accuracy was especially lower for samples from Slovakia and the USA. This was also already observed in case of the CC on country level (see Figure 4), where samples of the USA, but also Slovakia had a lower rate of correct classifications than samples from other geographical origins. The similarity of samples from the USA and Slovakia could also already be overserved in the PCA scores plot (see Figure 3).

With regard to separability using specific marker substances, by looking at the variable importance in RF classification, it was found that different markers were important, depending on which sample class was to be distinguished (see Table S7). Each marker was at least once among the top five ranked marker substances in all seven models, with the exception of TG (58:2) (best rank was 7th at Spanish samples vs. rest model). While the marker substances PE (34:1) and PC (36:5) did exhibit the best performance over all classification variants (median rank 6), the marker substances TG (58:2) and TG (60:3) were the least powerful (median rank 17). Especially, the marker substance PC (34:1) was an outstanding substance for the differentiation of more than one sample class.

With respect to lipid class, some distinct features were observed in the ranking of variable importance in the classification models: Marker substances belonging to the lipid class of phospholipids were particularly important in the classification models of samples from the Ukraine or the USA. DGs were potent marker substances for differentiating samples from Hungary from all other sample classes. In case of Spanish or French samples DGs were also mainly powerful for separation between samples from other countries. TGs were especially able to separate samples from Peru. Samples from Slovakia were most powerfully separated by a combination of phospholipids and TGs but also DGs, from all other samples in the dataset. These results indicate that especially the combined usage of marker substances from four lipid classes present in grain maize samples was an advantage to obtain high classification accuracy for all included sample classes.

## 4. Conclusions

The ability of analytical geographical origin verification based on the non-polar metabolome of grain maize samples by classification was investigated. For this purpose, 151 grain maize samples originating from seven countries worldwide were analyzed using UHPLC coupled to a high resolution qToF-analyzer. Lookup in metabolomics-related databases revealed that the non-polar metabolome of grain maize consisted mainly of acylglycerides and phospholipids. By multivariate data exploration, differences between the origin-specific sample groups could be observed and 20 high-potential marker substances were discovered, which were at the same time significant, reliable and high abundant. Those marker substances were further verified by LC-MS/MS and belonged to lipid species of PCs, PEs, DGs and TGs. Classification of all samples using peak areas of the identified 20 marker substances was found to provide a classification ability of 90.5% by RF algorithm. Classification of 20 different grain maize varieties grown at the same location did not exhibit lower classification accuracies than samples from other geographical origins. Additionally, the binary differentiation of samples from every country in the dataset from all other samples could be conducted by RF classification with high accuracies (89–99%), strengthening the high potential of the identified lipid marker substances for later usage in an authentication method.

However, as the differences in metabolite levels might be affected by factors which may vary from one year to another, it further has to be taken into account that the long-time stability of the metabolite profile of grain maize should be accessed in future studies. Also, it might be useful to extend the classification model in the future with samples originating from China. China was as of 2019/20 the biggest producing country of maize (over 260 million tons, 19% of world production) in the world despite most of its corn production is yet used in the country itself [21].

## Figures and Tables

**Table 1:** Classification results of two class RF models (one country vs. rest, 100 repeated 10-fold CV) using only the 20 identified marker substances, after pareto scaling as preprocessing.

Model	Accuracy ^1^
France vs. rest	98.9 ± 0.6%
Peru vs. rest	97.0 ± 0.4%
Slovakia vs. rest	91.5 ± 0.6%
Spain vs. rest	96.1 ± 0.6%
Ukraine vs. rest	98.5 ± 0.3%
Hungary vs. rest	99.2 ± 0.3%
USA vs. rest	89.3 ± 1.0%

^1^ Mean over 100 repeats along with standard deviation.

## Data Availability

The data presented in this study will be openly available in EBI/MetaboLights repository (https://www.ebi.ac.uk/metabolights, accessed on 6 September 2021) under the accession number “MTBLS3431”.

## Supplementary Materials

The following data are available online at https://www.mdpi.com/article/10.3390/foods10092160/s1, Table S1: Detailed metadata regarding the geographical origin of the grain maize sample set; Table S2: Grain maize varieties of samples from Spain (one sample per variety); Table S3: MANOVA test statistic results; Table S4: Additional information about the high potential marker substances, which were used for verification of the geographical sample origin. The substance identity was additionally verified by the MS/MS-fragmentation pattern (see Table S5); Table S5: Identification of marker substances suitable for an origin verification of grain maize samples by means of LC-MS/MS analysis; Table S6: Mahalanobis distances matrix. Only the selected and identified 20 metabolites were used (after pareto scaling) for calculation; Table S7: Ranking of variables according to the variable importance in binary Random Forest classification models of grain maize samples (one country vs. all other countries in the dataset). Figure S1: Survey view (*x*-axis: retention time; *y*-axis: *m*/*z*-values; Intensity: color-coded) of a representative LC-MS analysis of grain maize quality control sample; Figure S2: Canonical variate analysis scores plot. All 1022 metabolites were used (after pareto scaling) for calculation.; Figure S3: Heatmap of LSD-test *p*-values. The color-gradient is blue (at *p* = 0) over yellow (at *p* = 0.01, significance level border) to white (at *p* = 1); Figure S4: Canonical variate analysis scores plot. Only the selected and identified 20 metabolites were used (after pareto scaling) for calculation.

## Appendix A

Consensus peak table of all 1022 features in all 151 samples of the grain maize sample set.

## Appendix B

Peak areas of the selected 20 high performant lipid markers for geographic origin discrimination in all 151 samples of the grain maize sample set, as well as 10 replicates of the QC sample.
