# Supplementary material for: Non-Targeted LC-MS Metabolomics Approach towards an Authentication of the Geographical Origin of Grain Maize (Zea mays L.) Samples
Source: Foods. 2021 Sep 13;10(9):2160. doi: 10.3390/foods10092160 (PMC8466891; doi:10.3390/foods10092160)
Supplement: Supplementary file 1 [file foods-10-02160-s001.zip › foods-1357905-supplementary.pdf]

Supplementary Materials

Non-targeted LC-MS metabolomics approach towards an authentication of the geographical origin of grain maize (*Zea mays* L.) samples

David Schütz [1], Elisabeth Achten [2], Marina Creydt [1], Janet Riedl [2], Markus Fischer [1,*]

[1] Hamburg School of Food Science, Institute of Food Chemistry, University of Hamburg, Grindelallee 117, 20146 Hamburg, Germany; david.schuetz@uni-hamburg.de (D.S.); marina.creydt@uni-hamburg.de (M.C.);
[2] German Federal Institute for Risk Assessment (BfR), Department Safety in the Food Chain, Max-Dohrn-Str. 8-10, 10589 Berlin, Germany; elisabeth.achten@gmail.com (E.A.); janet.riedl@bfr.bund.de (J.R.)
* Correspondence: markus.fischer@uni-hamburg.de

Table S1. Detailed metadata regarding the geographical origin of the grain maize sample set.

Sample ID	Continent	Country	Region
1	Europe	France	Alsace
2	Europe	France	Alsace
3	Europe	France	Alsace
4	Europe	France	Alsace
6	Europe	France	Alsace
7	Europe	France	Alsace
8	Europe	France	Alsace
9	Europe	France	Alsace
10	Europe	France	Alsace
11	Europe	France	Alsace
12	Europe	France	Alsace
13	Europe	France	Alsace
14	Europe	France	Alsace
15	Europe	France	Alsace
16	Europe	France	Alsace
17	Europe	France	Alsace
18	Europe	France	Alsace
19	Europe	France	Alsace
20	Europe	France	Alsace
21	Europe	France	Alsace
22	Europe	France	Alsace
23	Europe	France	Alsace
24	Europe	France	Alsace
25	Europe	France	Alsace
26	Europe	France	Alsace
27	Europe	Hungary	NA
28	Europe	Hungary	NA
29	Europe	Hungary	NA
30	Europe	Hungary	NA
31	Europe	Hungary	NA
32	Europe	Hungary	NA
33	Europe	Hungary	NA
34	Europe	Hungary	NA
35	Europe	Hungary	NA
36	Europe	Hungary	NA
37	Europe	Hungary	NA
38	Europe	Hungary	NA
39	Europe	Hungary	NA
40	Europe	Hungary	NA
41	Europe	Hungary	NA
42	Europe	Hungary	NA
43	Europe	Hungary	NA
44	Europe	Hungary	NA
45	Europe	Hungary	NA
46	Europe	Hungary	NA
47	Europe	Hungary	NA
48	Europe	Hungary	NA

[1] NA = not available, USA = United States of America.

Table S1. *Cont.*

Sample ID	Continent	Country	Region
49	Europe	Hungary	NA
50	Europe	Hungary	NA
51	Europe	Spain	LLeida
52	Europe	Spain	LLeida
53	Europe	Spain	LLeida
54	Europe	Spain	LLeida
55	Europe	Spain	LLeida
56	Europe	Spain	LLeida
57	Europe	Spain	LLeida
58	Europe	Spain	LLeida
59	Europe	Spain	LLeida
60	Europe	Spain	LLeida
61	Europe	Spain	LLeida
62	Europe	Spain	LLeida
63	Europe	Spain	LLeida
64	Europe	Spain	LLeida
65	Europe	Spain	LLeida
66	Europe	Spain	LLeida
67	Europe	Spain	LLeida
68	Europe	Spain	LLeida
69	Europe	Spain	LLeida
70	Europe	Spain	LLeida
71	Europe	Ukraine	NA
72	Europe	Ukraine	NA
73	Europe	Ukraine	NA
74	Europe	Ukraine	NA
75	Europe	Ukraine	NA
76	Europe	Ukraine	NA
77	Europe	Ukraine	NA
78	Europe	Ukraine	NA
79	Europe	Ukraine	NA
80	Europe	Ukraine	NA
81	Europe	Ukraine	NA
82	Europe	Ukraine	NA
83	Europe	Ukraine	NA
84	Europe	Ukraine	NA
85	Europe	Ukraine	NA
86	Europe	Ukraine	NA
87	Europe	Ukraine	NA
88	Europe	Ukraine	NA
89	Europe	Ukraine	NA
90	Europe	Slovakia	Levice
91	Europe	Slovakia	Levice
92	Europe	Slovakia	Levice
93	Europe	Slovakia	Levice
94	Europe	Slovakia	Levice
95	Europe	Slovakia	Levice

[1] NA = not available, USA = United States of America.

Table S1. *Cont.*

Sample ID	Continent	Country	Region
96	Europe	Slovakia	Levice
97	Europe	Slovakia	Levice
98	Europe	Slovakia	Trnava
99	Europe	Slovakia	NA
100	Europe	Slovakia	Levice
101	Europe	Slovakia	Levice
102	Europe	Slovakia	Trnava
103	Europe	Slovakia	Senec
104	North America	USA	Alabama
105	North America	USA	Alabama
106	North America	USA	Alabama
107	North America	USA	Alabama
108	North America	USA	Arkansas
109	North America	USA	Arkansas
110	North America	USA	Arkansas
111	North America	USA	Arkansas
112	North America	USA	Georgia
113	North America	USA	Georgia
114	North America	USA	Georgia
115	North America	USA	Georgia
116	North America	USA	Louisiana
117	North America	USA	Louisiana
118	North America	USA	Louisiana
119	North America	USA	Louisiana
120	North America	USA	Mississippi
121	North America	USA	Mississippi
122	North America	USA	Mississippi
123	North America	USA	Mississippi
124	North America	USA	Texas
125	North America	USA	Texas
126	North America	USA	Texas
127	South America	Peru	Lambayeque
128	South America	Peru	Lambayeque
129	South America	Peru	Lambayeque
130	South America	Peru	Lambayeque
131	South America	Peru	Lambayeque
132	South America	Peru	Lambayeque
133	South America	Peru	Lambayeque
134	South America	Peru	Lambayeque
135	South America	Peru	Lambayeque
136	South America	Peru	Lambayeque
137	South America	Peru	Lambayeque
138	South America	Peru	Lambayeque
139	South America	Peru	Lambayeque
140	South America	Peru	Lambayeque
141	South America	Peru	Lambayeque
142	South America	Peru	Lambayeque

[1] NA = not available, USA = United States of America.

Table S1. *Cont.*

Sample ID	Continent	Country	Region
143	South America	Peru	Lambayeque
144	South America	Peru	Lambayeque
145	South America	Peru	Lambayeque
146	South America	Peru	Lambayeque
147	South America	Peru	Lambayeque
148	South America	Peru	Lambayeque
149	South America	Peru	Lambayeque
150	South America	Peru	Lambayeque
151	South America	Peru	Lambayeque

[1] NA = not available, USA = United States of America.

Table S2. Grain maize varieties of samples from Spain (one sample per variety).

68.K	Charleston	DKC6728	DKC6650	DKC5830
Ixabel	Kefieros	Keridos	Kontigos	LG30.600
LG30.681	MAS 64.P	MAS 66.C	MAS 75.A	Miloxan
Nystar	P1524	P1570	P1574	P1921

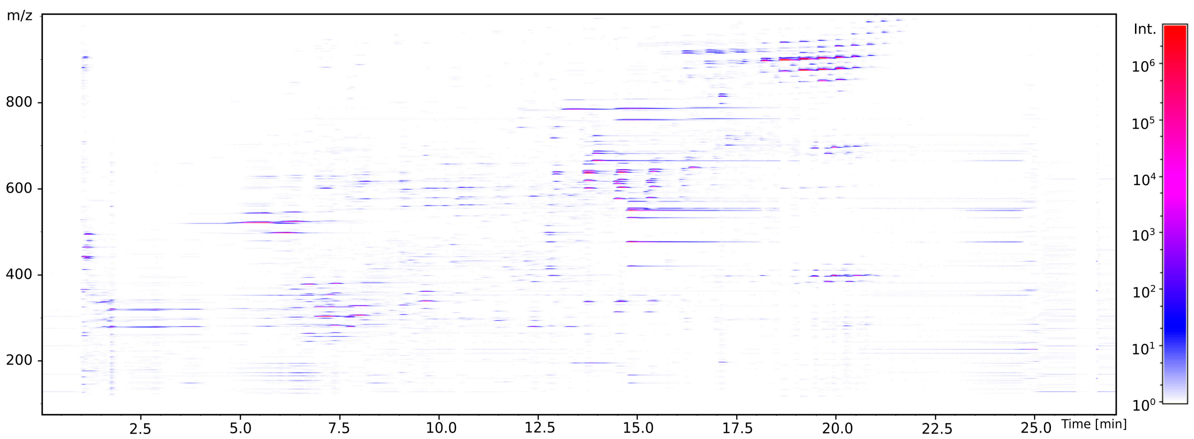

Figure S1. Survey view (x-axis: retention time; y-axis: *m*/*z*-values; Intensity: color-coded) of a representative LC-MS analysis of grain maize quality control sample.

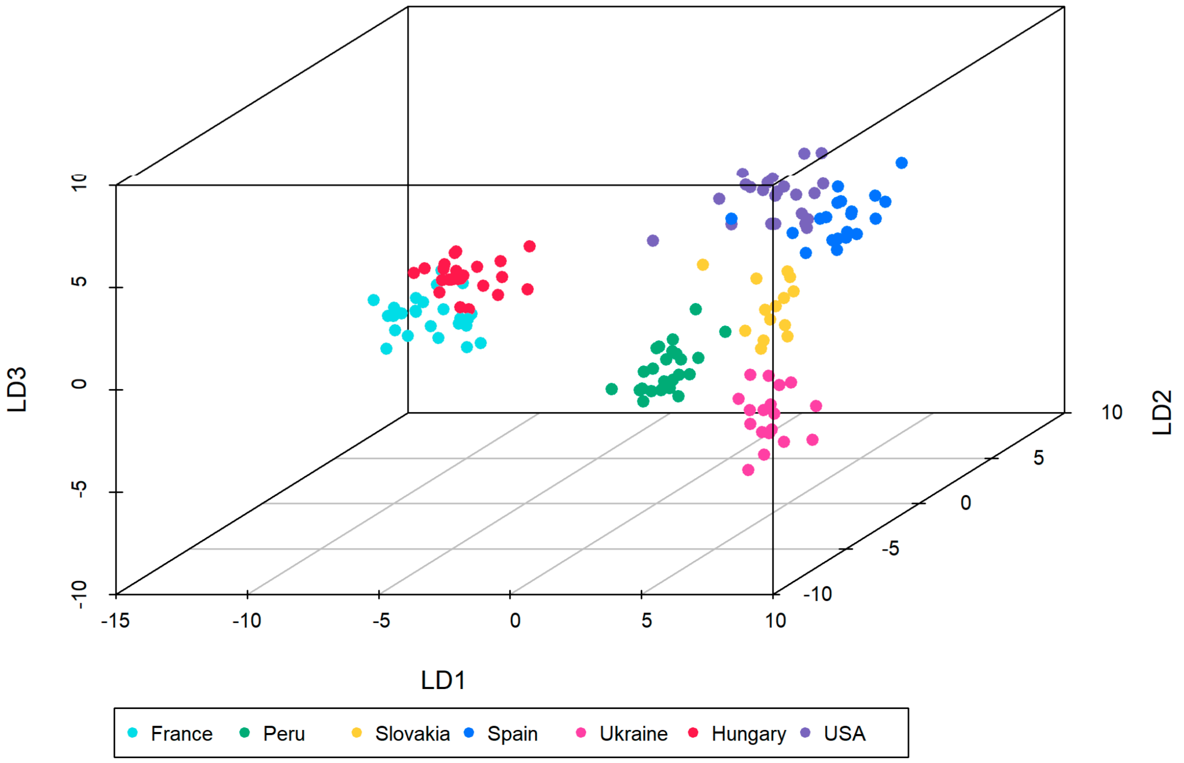

Figure S2. Canonical variate analysis scores plot. All 1022 metabolites were used (after pareto scaling) for calculation.

Table S3. MANOVA test statistic results.

	**Wilk's λ**	**F-value**	**num. Df**	**den. Df**	***p*-value**
1022 features	0.81422	-	3	147	1.186E-06
- PC1	-	0.4107	-	-	0.5226
- PC2	-	16.097	-	-	9.495E-05
- PC3	-	13.937	-	-	0.0002684
20 selected marker	0.80866	-	3	147	7.267E-07
- PC1	-	12.515	-	-	0.0005385
- PC2	-	9.0271	-	-	0.003122
- PC3	-	8.9612	-	-	0.003229

Table S4. Additional information about the high potential marker substances, which were used for verification of the geographical sample origin. The substance identity was additionally verified by the MS/MS-fragmentation pattern (see Table S5).

Retention time [min]	*m*/*z*-value [Da]	Compound	Adduct	ANOVA *p*-value	Counts of significant pairings in Tukey test	Relative standard deviation in QC-samples	Max. peak area observed
12.9	716.523	PE (34:2)	$[M+H]^+$	5.94E-24	15	5.1%	1.35E+07
13.9	718.537	PE (34:1)	$[M+H]^+$	2.47E-20	10	4.0%	1.83E+06
13.0	742.538	PE (36:3)	$[M+H]^+$	2.71E-18	12	7.3%	5.52E+06
16.5	760.586	PC (34:1)	$[M+H]^+$	4.97E-21	12	4.7%	7.29E+07
12.3	780.553	PC (36:5)	$[M+H]^+$	5.09E-39	16	6.3%	2.97E+06
14.6	784.586	PC (36:3)	$[M+H]^+$	3.66E-27	14	4.2%	1.10E+08
15.3	586.540	DG (32:0)	$[M+NH_4]^+$	1.59E-41	15	9.3%	1.24E+06
14.6	610.541	DG (34:2)	$[M+NH_4]^+$	5.25E-38	15	3.1%	4.22E+07
15.4	612.556	DG (34:1)	$[M+NH_4]^+$	1.35E-39	16	2.6%	2.45E+07
13.0	632.525	DG (36:5)	$[M+NH_4]^+$	8.38E-41	16	4.1%	1.00E+07
16.2	640.588	DG (36:1)	$[M+NH_4]^+$	2.86E-35	16	3.3%	4.27E+06
18.3	868.740	TG (52:6)	$[M+NH_4]^+$	7.28E-41	12	4.5%	1.71E+06
19.4	886.788	TG (53:4)	$[M+NH_4]^+$	1.88E-25	13	6.3%	3.20E+06
20.5	930.852	TG (56:3)	$[M+NH_4]^+$	6.12E-29	13	6.4%	1.10E+07
20.9	932.868	TG (56:2)	$[M+NH_4]^+$	1.92E-32	11	7.1%	9.70E+06
20.8	958.883	TG (58:3)	$[M+NH_4]^+$	5.75E-28	10	6.1%	1.88E+06
21.2	960.898	TG (58:2)	$[M+NH_4]^+$	1.76E-25	9	5.7%	3.22E+06
21.6	962.913	TG (58:1)	$[M+NH_4]^+$	8.72E-41	10	5.7%	1.70E+06
21.2	986.914	TG (60:3)	$[M+NH_4]^+$	5.46E-36	8	6.6%	3.29E+06
21.6	988.929	TG (60:2)	$[M+NH_4]^+$	2.36E-37	8	4.9%	3.10E+06

	PE (34:2)	PE (34:1)	PE (36:3)	PC (34:1)	PC (36:5)	PC (36:3)	DG (32:0)	DG (34:2)	DG (34:1)	DG (36:5)	DG (36:1)	TG (52:6)	TG (53:4)	TG (56:3)	TG (56:2)	TG (58:3)	TG (58:2)	TG (58:1)	TG (60:3)	TG (60:2)
**France - Peru**	0.0064	0.0000	1.0000	1.0000	0.0000	0.0880	0.0000	0.0000	0.0000	0.0000	0.0000	0.0000	0.0001	0.0000	0.0000	0.0000	0.0000	0.0000	0.0000	0.0000
**France - Slovakia**	1.0000	0.5188	1.0000	1.0000	0.0009	1.0000	0.0000	0.0000	0.0000	0.0000	0.0000	0.0000	0.0000	0.0996	1.0000	1.0000	1.0000	1.0000	1.0000	1.0000
**France - Spain**	0.0009	0.0000	0.0000	0.0000	0.0000	0.0000	0.0000	0.0000	0.0000	0.0000	0.0000	0.0000	0.0043	0.0000	0.1118	0.0224	0.2680	1.0000	1.0000	1.0000
**France - Ukraine**	0.0000	0.2030	0.0013	0.0010	0.0000	0.0000	0.0000	0.0358	0.0054	0.0193	0.0000	0.0000	0.0000	0.0047	1.0000	1.0000	0.6330	1.0000	1.0000	1.0000
**France - Hungary**	0.0004	0.0000	0.0000	0.0212	0.0000	0.0000	1.0000	0.0035	1.0000	1.0000	1.0000	1.0000	0.7211	0.4810	0.0114	0.0019	0.0068	0.0007	0.0322	0.0120
**France - USA**	1.0000	0.0000	0.4518	0.0000	0.0000	0.0017	0.0000	0.0000	0.0000	0.0000	0.0000	0.0000	0.2840	0.0005	1.0000	1.0000	1.0000	0.0005	1.0000	1.0000
**Peru - Slovakia**	0.0053	0.8401	1.0000	1.0000	0.0000	0.0060	1.0000	1.0000	0.0000	1.0000	0.0000	0.0713	0.0000	0.0000	0.0000	0.0000	0.0000	0.0000	0.0000	0.0000
**Peru - Spain**	0.0000	0.1032	0.0003	0.0000	0.0000	0.0000	0.0000	0.0125	0.0210	0.0000	1.0000	0.1492	0.0000	0.0000	0.0000	0.0000	0.0000	0.0000	0.0000	0.0000
**Peru - Ukraine**	0.0071	0.0000	0.0000	0.0000	1.0000	0.0053	0.0164	0.0000	0.0000	0.0000	0.0000	0.0000	0.0000	0.0000	0.0000	0.0000	0.0000	0.0000	0.0000	0.0000
**Peru - Hungary**	0.0000	1.0000	0.0000	0.5901	0.0000	0.0000	0.0000	0.0000	0.0000	0.0000	0.0000	0.0000	0.1372	0.0000	0.0000	0.0000	0.0000	0.0000	0.0000	0.0000
**Peru - USA**	0.0017	1.0000	1.0000	0.0000	0.0000	0.0000	1.0000	0.0683	0.0000	1.0000	0.0000	1.0000	0.0000	0.0000	0.0000	0.0000	0.0000	0.0000	0.0000	0.0000
**Slovakia - Spain**	0.0572	0.0004	0.0299	0.0004	0.0002	0.0063	0.0001	0.0023	0.0069	0.0000	0.0017	1.0000	1.0000	1.0000	1.0000	0.0831	1.0000	1.0000	1.0000	1.0000
**Slovakia - Ukraine**	0.0000	0.0004	0.0000	0.0001	0.0000	0.0000	0.0102	0.0022	0.3897	0.0000	1.0000	0.6684	1.0000	1.0000	1.0000	1.0000	1.0000	1.0000	1.0000	1.0000
**Slovakia - Hungary**	0.0435	0.2729	0.0110	1.0000	1.0000	0.0293	0.0000	0.0000	0.0000	0.0000	0.0000	0.0000	0.0000	0.0001	0.0022	0.0214	0.0143	1.0000	0.1333	0.1247
**Slovakia - USA**	1.0000	0.0329	1.0000	0.0009	1.0000	0.5755	1.0000	1.0000	1.0000	0.7399	1.0000	1.0000	0.0384	1.0000	1.0000	1.0000	1.0000	1.0000	1.0000	1.0000
**Spain - Ukraine**	0.0000	0.0000	0.0000	0.0000	0.0000	0.0000	0.0000	0.0000	0.0000	0.0000	0.0000	0.0828	0.0611	1.0000	1.0000	1.0000	1.0000	1.0000	1.0000	1.0000
**Spain - Hungary**	1.0000	0.4076	1.0000	0.0127	0.0120	1.0000	0.0000	0.0000	0.0000	0.0000	0.0000	0.0000	0.0000	0.0000	0.0000	0.0000	0.0000	0.0374	0.0009	0.0075
**Spain - USA**	0.0058	1.0000	0.0240	1.0000	0.0000	1.0000	0.0000	0.0000	0.0000	0.0000	0.0001	1.0000	1.0000	1.0000	1.0000	0.1421	0.1371	0.0241	1.0000	1.0000
**Ukraine - Hungary**	0.0000	0.0000	0.0000	0.0000	0.0000	0.0000	0.0000	0.0000	0.0000	0.0009	0.0000	0.0000	0.0000	0.0000	0.0004	0.0000	0.0000	0.0013	0.0003	0.0008
**Ukraine - USA**	0.0000	0.0000	0.0000	0.0000	0.0000	0.0000	1.0000	0.1156	1.0000	0.0056	1.0000	0.0026	0.0003	1.0000	1.0000	1.0000	0.3415	0.0008	1.0000	0.2232
**Hungary - USA**	0.0032	1.0000	0.0067	0.0317	1.0000	1.0000	0.0000	0.0000	0.0000	0.0000	0.0000	0.0000	0.0002	0.0000	0.0001	0.0003	0.0251	1.0000	0.0849	1.0000

Figure S3. Heatmap of LSD-test *p*-values. The color-gradient is blue (at $p = 0$) over yellow (at $p = 0.01$, significance level border) to white (at $p = 1$).

Table S5. Identification of marker substances suitable for an origin verification of grain maize samples by means of LC-MS/MS analysis.

Compound	Sum formula	Adduct	*m*/*z*-value calculated [Da]	*m*/*z*-value observed [Da]	Error [ppm]	Relevant MS/MS-fragments	Retention time [min]
PE (18:2/16:0)	$C_{39}H_{74}NO_8P$	$[M+H]^+$	716.5225	716.5229	1	575.5 ($[M-141+H]^+$, loss of phosphatidylethanolamine head group), 337.3 ($[RC=O+74]^+$, acylium ion of FA(18:2))	12.9
PE (18:1/16:0)	$C_{39}H_{76}NO_8P$	$[M+H]^+$	718.5381	718.5374	1	577.5 ($[M-141+H]^+$, loss of phosphatidylethanolamine head group), 339.3 ($[RC=O+74]^+$, acylium ion of FA(18:1))	13.9
PE (18:1/18:2)	$C_{41}H_{76}NO_8P$	$[M+H]^+$	742.5381	742.5383	< 1	601.5 ($[M-141+H]^+$, loss of phosphatidylethanolamine head group), 339.3 ($[RC=O+74]^+$, acylium ion of FA(18:1))	13.0
PC (34:1)	$C_{42}H_{82}NO_8P$	$[M+H]^+$	760.5851	760.5859	1	577.5 ($[M-183+H]^+$, loss of phosphocholine head group), 184.1 ($[C_5H_{15}NO_4P]^+$, phosphocholine head group)	16.5
PC (36:5)	$C_{44}H_{78}NO_8P$	$[M+H]^+$	780.5538	780.5532	1	597.5 ($[M-183+H]^+$, loss of phosphocholine head group), 184.1 ($[C_5H_{15}NO_4P]^+$, phosphocholine head group)	12.3
PC (36:3)	$C_{44}H_{82}NO_8P$	$[M+H]^+$	784.5851	784.5862	1	601.5 ($[M-183+H]^+$, loss of phosphocholine head group), 184.1 ($[C_5H_{15}NO_4P]^+$, phosphocholine head group)	14.6
DG (16:0/16:0)	$C_{35}H_{68}O_5$	$[M+NH_4]^+$	586.5405	586.5396	2	313.3 ($[RC=O+74]^+$, acylium ion of FA(16:0))	15.3
DG (18:2/16:0)	$C_{37}H_{68}O_5$	$[M+NH_4]^+$	610.5405	610.5408	< 1	337.3, 313.3 ($[RC=O+74]^+$, acylium ions of FA(18:2) resp. FA(16:0))	14.6
DG (18:1/16:0)	$C_{37}H_{70}O5$	$[M+NH_4]^+$	612.5561	612.5561	< 1	339.3, 313.3 ($[RC=O+74]^+$, acylium ions of FA(18:1) resp. FA(16:0))	15.4
DG (18:2/18:3)	$C_{39}H_{66}O_5$	$[M+NH_4]^+$	632.5248	632.5245	1	337.3, 335.3 ($[RC=O+74]^+$, acylium ions of FA(18:2) resp. FA(18:1))	13.0
DG (18:0/18:1)	$C_{39}H_{74}O_5$	$[M+NH_4]^+$	640.5874	640.5875	< 1	341.3, 339.3 ($[RC=O+74]^+$, acylium ions of FA(18:0) resp. FA(18:1))	16.2

[1] FA = fatty acid

Table S5. *Cont.*

Compound	Sum formula	Adduct	*m/z*-value calculated [Da]	*m/z*-value observed [Da]	Error [ppm]	Relevant MS/MS-fragments	Retention time [min]
TG (16:1/18:3/18:2)	$C_{55}H_{94}O_6$	$[M+NH_4]^+$	868.7389	868.7401	1	597.5, 573.5, 571.5 (neutral loss of $[RCOOH+NH_3]$ with RCOOH = FA(16:1), FA(18:3) resp. FA(18:2))	18.3
TG (17:0/18:2/18:2)	$C_{56}H_{100}O_6$	$[M+NH_4]^+$	886.7858	886.7881	3	599.5, 589.5 (neutral loss of $[RCOOH+NH_3]$ with RCOOH = FA(17:0) resp. FA(18:2))	19.4
TG (18:2/18:1/20:0)	$C_{59}H_{108}O_6$	$[M+NH_4]^+$	930.8484	930.8515	3	633.6, 631.6, 601.5 (neutral loss of $[RCOOH+NH_3]$ with RCOOH = FA(18:2), FA(18:1) resp. FA(20:0))	20.5
TG (18:1/18:1/20:0)	$C_{59}H_{110}O_6$	$[M+NH_4]^+$	932.8641	932.8677	4	633.6, 603.5 (neutral loss of $[RCOOH+NH_3]$ with RCOOH = FA(18:1), resp. FA(20:0))	20.9
TG (18:2/18:1/22:0)	$C_{61}H_{112}O_6$	$[M+NH_4]^+$	958.8797	958.8827	3	661.6, 659.6, 601.5 (neutral loss of $[RCOOH+NH_3]$ with RCOOH = FA(18:2), FA(18:1) resp. FA(22:0))	20.8
TG (16:1/18:1/24:0)	$C_{61}H_{114}O_6$	$[M+NH_4]^+$	960.8954	960.8983	3	689.6, 661.6, 575.5 (neutral loss of $[RCOOH+NH_3]$ with RCOOH = FA(16:1), FA(18:1) resp. FA(24:0))	21.2
TG (16:0/18:1/24:0)	$C_{61}H_{116}O_6$	$[M+NH_4]^+$	962.9116	962.9128	2	689.7, 663.6, 577.5 (neutral loss of $[RCOOH+NH_3]$ with RCOOH = FA(16:0), FA(18:1) resp. FA(24:0))	21.6
TG (18:2/18:1/24:0)	$C_{63}H_{116}O_6$	$[M+NH_4]^+$	986.9110	986.9139	3	689.6, 687.6, 601.5 (neutral loss of $[RCOOH+NH_3]$ with RCOOH = FA(18:2), FA(18:1) resp. FA(24:0))	21.2
TG (18:1/18:1/24:0)	$C_{63}H_{118}O_6$	$[M+NH_4]^+$	988.9267	988.9287	2	689.6, 603.5 (neutral loss of $[RCOOH+NH_3]$ with RCOOH = FA(18:1), resp. FA(24:0))	21.6

[1] FA = fatty acid

Table S6. Mahalanobis distances matrix. Only the selected and identified 20 metabolites were used (after pareto scaling) for calculation.

	**France**	**Peru**	**Slovakia**	**Spain**	**Ukraine**	**Hungary**	**USA**
France	0	33143	15216	27156	31850	24706	19455
Peru	33143	0	28009	37469	21789	53614	34507
Slovakia	15216	28009	0	16710	32354	31348	16515
Spain	27156	37469	16710	0	47245	31232	13774
Ukraine	31850	21789	32354	47245	0	56029	42154
Hungary	24706	53614	31348	31232	56029	0	26093
USA	19455	34507	16516	13774	42154	26903	0

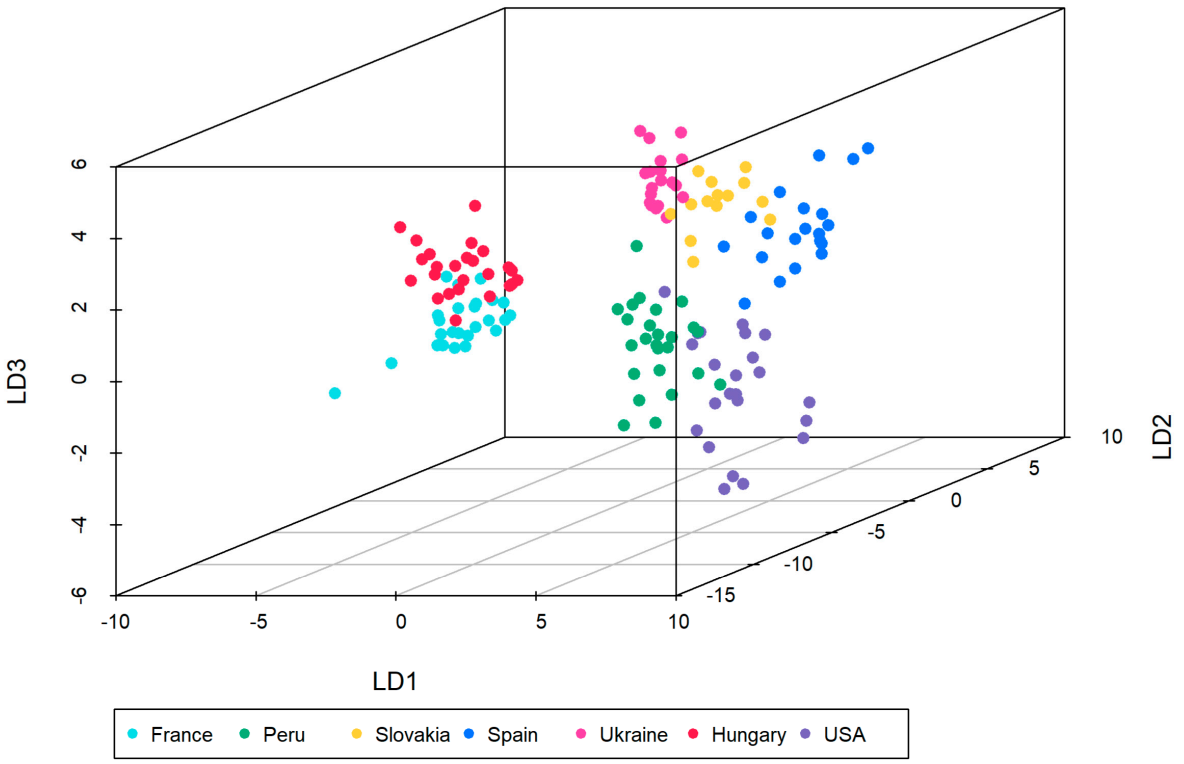

Figure S4. Canonical variate analysis scores plot. Only the selected and identified 20 metabolites were used (after pareto scaling) for calculation.

Table S7. Ranking of variables according to the variable importance in binary Random Forest classification models of grain maize samples (one country vs. all other countries in the dataset).

**Rank**	**France vs. rest**	**Peru vs. rest**	**Slovakia vs. rest**	**Spain vs. rest**	**Ukraine vs. rest**	**Hungary vs. rest**	**USA vs. rest**
1	PE (34:1)	TG (60:3)	TG (53:4)	DG (36:5)	PC (34:1)	DG (34:2)	PC (34:1)
2	TG (52:6)	TG (60:2)	PE (34:1)	DG (32:0)	PE (34:1)	DG (34:1)	PE (36:3)
3	DG (34:1)	TG (58:1)	PC (34:1)	DG (34:2)	PC (36:3)	DG (36:1)	PE (34:2)
4	DG (36:1)	TG (56:2)	TG (52:6)	PC (36:5)	PE (36:3)	DG (36:5)	TG (56:3)
5	DG (32:0)	TG (58:3)	DG (36:5)	TG (58:3)	PE (34:2)	DG (32:0)	PC (36:5)
6	DG (34:2)	TG (56:3)	PC (36:5)	TG (56:3)	PC (36:5)	PE (34:1)	TG (58:1)
7	TG (58:1)	DG (34:1)	DG (32:0)	TG (58:2)	TG (52:6)	PE (36:3)	DG (36:5)
8	DG (36:5)	TG (58:2)	TG (58:3)	TG (56:2)	TG (60:2)	TG (58:1)	PC (36:3)
9	PE (36:3)	PC (36:5)	PE (36:3)	PC (34:1)	TG (58:1)	TG (52:6)	DG (34:1)
10	PC (34:1)	DG (36:1)	DG (36:1)	TG (58:1)	TG (53:4)	PC (34:1)	PE (34:1)
11	PC (36:5)	PE (34:2)	DG (34:2)	PE (34:1)	TG (60:3)	TG (60:2)	DG (36:1)
12	PC (36:3)	TG (53:4)	TG (56:3)	PE (36:3)	DG (36:1)	TG (56:2)	TG (58:3)
13	TG (60:2)	PC (36:3)	PE (34:2)	TG (60:2)	DG (34:1)	TG (58:3)	DG (32:0)
14	TG (53:4)	DG (36:5)	TG (60:2)	PE (34:2)	TG (58:3)	TG (53:4)	TG (56:2)
15	TG (58:3)	PC (34:1)	TG (58:1)	DG (34:1)	DG (36:5)	PE (34:2)	DG (34:2)
16	PE (34:2)	PE (36:3)	DG (34:1)	PC (36:3)	TG (56:3)	PC (36:3)	TG (58:2)
17	TG (58:2)	DG (34:2)	TG (58:2)	TG (60:3)	DG (32:0)	TG (60:3)	TG (60:2)
18	TG (56:2)	TG (52:6)	PC (36:3)	DG (36:1)	TG (56:2)	TG (58:2)	TG (60:3)
19	TG (56:3)	DG (32:0)	TG (56:2)	TG (52:6)	TG (58:2)	PC (36:5)	TG (52:6)
20	TG (60:3)	PE (34:1)	TG (60:3)	TG (53:4)	DG (34:2)	TG (56:3)	TG (53:4)

R Code for data analysis.

```
#########################################################################################
# R function calls (R, version 3.6.3)                                                  #
# -----------------------------------------                                             #
# All examples expect the raw-data organized in a matrix "raw.data", formatted with one #
# sample per row and one marker metabolite per column.                                  #
# The sample classes have to be given in the factor "class.labels".                     #
#########################################################################################

#####################################################
# Univariate statistics                             #
#####################################################
# One-way ANOVA test
# -----------------------------
# Input: raw.data given in matrix "raw.data", sample classes given in factor "class.labels".
# Output: "ANOVA.pvalues" a vector of ANOVA p-values, one for each marker.
ANOVA.objects<-list()
ANOVA.pvalues<-c()
for (i in 1:ncol(raw.data)) {
 ANOVA.input<-data.frame(group = class.labels, marker = raw.data[,i])
 ANOVA.objects[[i]]<-aov(marker ~ group, data = ANOVA.input)
 ANOVA.pvalues<-c(ANOVA.pvalues, summary(ANOVA.objects[[i]])[[1]][1,5])
}

# post-hoc Tukey-test
# -------------------------
# Input: ANOVA-results given in list "ANOVA.objects".
# Output: "Tukey.pvalues" a matrix of Tukey-Test p-values, one column for each marker.
Tukey.pvalues<-matrix(NA, nrow = 21, ncol = ncol(raw.data))
colnames(Tukey.pvalues)<-paste("marker", c(1:ncol(raw.data)), sep=".")
for (i in 1:length(ANOVA.objects)) {
 Tukey.object<-TukeyHSD(ANOVA.objects[[i]], conf.level = 0.99)
 Tukey.pvalues[,i]<-Tukey.object$group[,4]
}
row.names(Tukey.pvalues)<-names(Tukey.object$group[,4])

# Least squared differences (LSD)
# ----------------------------------------
# Input: ANOVA-results given in list "ANOVA.objects".
# Output: "LSD.results" a list of data.frames with results of LSD, one for each marker.
#            "LSD.pvalues" a list of data.frames with p-values of LSD, one for each marker.
library(agricolae)  # version 1.3-5
LSD.results<-list()
for (i in 1:ncol(raw.data)) {
 LSD.object<-LSD.test(ANOVA.objects[[i]], "group", p.adj = "bonferroni", console = F, alpha = 0.01)
 LSD.results[[i]]<-cbind(group = row.names(LSD.object$groups),
                         LSD.indicators = as.character(LSD.object$groups$groups))
 LSD.results[[i]]<-LSD.results[[i]][order(LSD.results[[i]][,1]),]
}
```

```
LSD.pvalues<-list()
for (i in 1:ncol(raw.data)) {
  LSD.object<-LSD.test(ANOVA.objects[[i]], "group", p.adj = "bonferroni",
                                   console = F, alpha = 0.01, group = F)
  LSD.pvalues[[i]]<-cbind(group = row.names(LSD.object$comparison),
                                      LSD.pvalues = as.character(LSD.object$comparison$pvalues))
}


##############################################
# Preprocessing                                                       #
##############################################
# Pareto scaling of raw data
# ---------------------------------
# Input: raw.data given in matrix "raw.data".
# Output: "pareto.data" a matrix of the pareto-scaled data.
pareto.data<-matrix(NA, nrow = nrow(raw.data), ncol = ncol(raw.data))
colnames(pareto.data)<-colnames(raw.data)
for (i in 1:ncol(raw.data)) {
  col.Mean<-mean(raw.data[,i])
  col.Sqrt.SD<-sqrt(sd(raw.data[,i]))
  pareto.data[,i]<-((raw.data[,i] - col.Mean) / col.Sqrt.SD)
}

##############################################
# Multivariate data analysis                                    #
##############################################
# Principal component analysis (PCA)
# ---------------------------------------------
# Input: Pareto-scaled data given in matrix "pareto.data".
# Output: "pca.scores" a matrix of the PCA scores and "pca.loadings" a matrix of the PCA
#               loadings values.
pca.object<-prcomp(x = pareto.data, center = F, scale. = F)
pca.scores<-pca.object$x
pca.loadings<-pca.object$rotation

# Mahalanobis distances
# -----------------------------
# Input: Pareto-scaled data given in matrix "pareto.data".
# Output: "Mahalanobis.Distances" a matrix of the groupwise Mahalanobis distances.
library(HDMD)  # version 1.2
mahalanobis.object<-pairwise.mahalanobis(x = pareto.data, grouping = class.labels)
Mahalanobis.Distances<-sqrt(mahalanobis.object$distance)
colnames(Mahalanobis.Distances)<-names(mahalanobis.object$means[,1])
row.names(Mahalanobis.Distances)<-names(mahalanobis.object$means[,1])
```

```
# MANOVA test
# ------------------
# Input: "manova.input" data.frame of first three principal components of PCA and sample classes.
# Output: "manova.result" a summary of the MANOVA analysis / Wilk's test.
manova.input<-as.data.frame(cbind(Group = class.labels, pca.scores[,1:3]))
manova.object<-manova(cbind(PC1,PC2,PC3) ~ Group, data = manova.input)
manova.result<-summary(manova.object, test = "Wilks")
manova.details.result<-summary.aov(manova.object, test = "Wilks")

# Canonical variate analysis (CVA)
# ------------------------------------------
# Input: Pareto-scaled data given in matrix "pareto.data" and sample classes
#           given in factor "class.labels".
# Output: "cva.scores" the scores of the canonical variate analysis.
library(MASS)  # version 7.3-51.5
cva.object<-lda(x = pareto.data, grouping = class.labels)
cva.result<-predict(cva.object)
cva.scores<-cva.result$x[,1:3]

##############################################
# Classification                                                #
##############################################
# Random Forest classification (RF)
# ------------------------------------------
# 10-fold cross-validation, 100 times repeated
# Input: Pareto-scaled data given in matrix "pareto.data", sample classes given in factor "class.labels".
#           For calculation of "one-vs.-rest" models the parameter y, "class.labels", of the train function
#           has to be exchanged by "binary.class.labels[[i]]".
# Output: "rf.model" the Random Forest model. Classifiaction matrix in "rf.classification.matrix",
#              Accuracy in "rf.accuracy", standard deviation of accuracy over the 100 repeats
#              in "rf.AccuracySD.per.repeat" and variable importance in "rf.variable.importance".
binary.class.labels<-list()
group.names<-unique(as.character(class.labels))
for (i in 1:length(group.names)) {
  i.binary.class.labels<-as.character(class.labels)
  rest.samples<-which(i.binary.class.labels!=group.names[i])
  i.binary.class.labels[rest.samples]<-"rest"
  binary.class.labels[[i]]<-as.factor(i.binary.class.labels)
}
library(caret)  # version 6.0-86
library(randomForest)  # version 4.6-14
tuning.info<-expand.grid(mtry = trunc(sqrt(ncol(pareto.data))))
train.ctrl<-trainControl(method="repeatedcv", number = 10, repeats = 100, classProbs = T,
                          returnResamp = "all", summaryFunction = multiClassSummary,
                          savePredictions = T, allowParallel = T)
rf.model<-train(x = pareto.data, y = class.labels, method = "rf", ntree = 1000,
                 tuneGrid = tuning.info, trControl = train.ctrl)
rf.confusion.matrix<-confusionMatrix.train(rf.model, "none")
rf.accuracy<-sum(diag(rf.confusion.matrix$table))/(nrow(pareto.data)*100)
rf.variable.importance<-varImp(rf.model)$importance
# Standard deviation of model accuracy, measured per repeat
repeat.label<-paste("*.Rep", formatC(c(1:100), width = 3, flag = "0"), sep = "")
```

```
accuracy.per.repeat<-c()
for (i in 1:100) {
  # Identify repeat i in table of predictions "rf.model$pred"
  akt.repeat.subtable<-rf.model$pred[grep(pattern = repeat.label[i], x = rf.model$pred$Resample),]
  accuracy.per.repeat<-c(accuracy.per.repeat,
                         sum(diag(table(actual = akt.repeat.subtable$obs,
                         predictions = akt.repeat.subtable$pred))))
}
rf.AccuracySD.per.repeat<-(sd(accuracy.per.repeat)/nrow(akt.repeat.subtable))
```
